# Risk factors of ankle osteoarthritis in the treatment of critical bone defects using ilizarov technique

**DOI:** 10.1186/s12891-021-04214-8

**Published:** 2021-04-09

**Authors:** Kai Liu, Feiyu Cai, Yanshi Liu, Alimujiang Abulaiti, Peng Ren, Aihemaitijiang Yusufu

**Affiliations:** Department of Trauma and Microreconstructive Surgery, The First Affiliated Hospital of Xinjiang Medical University, 830054 Urumqi, Xinjiang China

**Keywords:** Bone defect, Bone transport, Ilizarov technique, Joint, Osteoarthritis

## Abstract

**Background:**

Distraction osteogenesis using the Ilizarov external circular fixator has been applied in lower limb reconstructive surgery widely. The increasing ankle osteoarthritis (OA) progression and severity are often associated with the period of external fixator and the greater relative instability of the ankle joint, but few studies have quantified risk factors directly during this technique.

**Methods:**

The study was conducted on 236 patients who underwent bone transport surgery for tibias using the Ilizarov external circular fixator from 2008 to 2018. The cumulative incidence of ankle OA diagnoses in patients after the Ilizarov technique treatment was calculated and stratified by risk factors from preoperative and postoperative management. After the data were significant through the Mann-Whitney U test analyzed, odds ratios were calculated using logistic regression to describe factors associated with the OA diagnosis including gender, age, BMI, location of bone defect, diabetes, hypertension, osteoporosis, the history of metal allergy and glucocorticoid intake, the American Orthopaedic Foot & Ankle Society (AOFAS) ankle-HF scale scores, defect size (DS), the type of bone transport, the bone union time, external fixator time (EFT), and external fixator index (EFI).

**Results:**

There were 199 males and 37 females with a mean age of 47 years (range 28–59 years). Out of 236 patients, 49 had an additional treatment for ankle OA after the Ilizarov technique treatment of bone defects (average follow-up time 2.1 years, range 1.6–4.2 years). The incidence of postoperative ankle OA was 20.8 %, with 19 patients classified as K&L grade 3 and seven patients as grade 4. The top five risk factors included double-level bone transport (OR3.79, *P* = 0.005), EFI > 50days/cm (OR3.17, *P* = 0.015), age > 45years (OR2.29, *P* = 0.032), osteoporosis (OR1.58, *P* < 0.001), BMI > 25 (OR1.34, *P* < 0.001). Male, BMI > 25, diabetes, osteoporosis, and AOFAS ankle-HF scale scores are the independent risk factors.

**Conclusions:**

Ilizarov external circular fixator is a safe and effective method of treatment for critical bone defects. The double level bone transport, EFI > 50days/cm, age > 45years, osteoporosis, BMI > 25 are the top five relevant risk factors of ankle OA. The probability of developing ankle OA among patients having three or more risk factors is 50–70 %.

## Background

Ilizarov external circular fixator has been an established technique for treating critical bone defects (> 4 cm) caused by trauma, tumor resection, and osteomyelitis debridement widely [1–7]. However, its complications such as pin tract infections, joint stiffness, and especially osteoarthritis (OA), have also been becoming an obstacle to postoperative rehabilitation gradually [8, 9]. In comparison with other joints, bone defects in the lower limb of the body commonly result in complications of knee and ankle OA, during the long period of the bone defect reconstruction using the external fixator. [3, 10]. In the part of the tibia, the rates of arthritis cases up to 12 % [11] are mostly caused by the anatomical structure and poor perfusion in the distal third of the leg [11]. Although knee OA is dominant, the ankle joint is fixed by high-strength, long-term maintenance to provide stable support for bone transport, which nourished the OA symptom too. In fact, the patient is encouraged to start exercises and joint rehabilitation training earlier to prevent the stiffness and deterioration of joints after the operation. However, the nature of the complication combined with the limitations in the external fixator results in patients suffering from ankle OA and requiring surgical intervention again. This not only decreases the quality of life for the patient but also poses a significant societal burden.

Despite joint injury is a critical reason for the onset of ankle OA, a way to prevent its progression following these injuries remains elusive. Based on historical consensus, the diagnosis of ankle OA can be based on X-ray characteristics, and the Kellgren and Lawrence (K&L) scale is the accepted reference standard selected by the World Health Organization [12]. Via recent research, the major reason for ankle OA is the complexity of how cartilage interacts and responds to external factors. Also, it is becoming more obvious that the progress of ankle OA is the result of a combination of biological, mechanical, and structural factors [13]. These three factors work in unity to maintain cartilage homeostasis. However, it should not be underestimated that the epidemics factors and metabolic diseases that lead to ankle cartilage degeneration and OA symptoms further since the Ilizarov technique for the treatment of tibia bone defects.

Some authors who reported ankle OA rates considered them mostly as an internal fixator complication, with no vital impact on external fixator treatment outcomes [7, 14–18]. Thus, there are rare researches on the risk factors of ankle OA, a severe of complications in the treatment of the tibia bone defect treated with the Ilizarov technique. The purpose of this research was to review the incidence of external fixator complication which progress to ankle OA, examine the current understanding of the mechanisms thought to be responsible for ankle OA, and evaluate the current risk factors of ankle OA during the Ilizarov technique in the treatment of bone defects.

## Methods

This study was a retrospective assessment of 236 patients treated by the same surgery team for bone defects of the tibia between 2008 and 2018. The inclusion criteria were bone defects of the tibia at least 4 cm managed definitively using an external circular fixator of the Ilizarov technique, at least 2 years after treatment ends, observer access to all medical records, and radiological images relating to the patients’ treatment. Other patients older than 60 years, with a poor compliance or any other diseases that can postpone bone healing (such as dermatitis, tuberculosis, autoimmune diseases, kidney disease, etc.) were excluded. 199 men and 37 women were assessed in this study according to the inclusion criteria. Bone defects were caused by trauma in 87 cases, tumor resection in 10 cases, and osteomyelitis debridement in 139 cases. Bone defects between 4.5 and 6 cm were managed with single-level bone transport,and more than 6.0 cm with double-level bone transport. The study was conducted by the Declaration of Helsinki, approved by our hospital institutional review board. The analyzed medical and radiological data came from hospital records.

### Surgical technique

At the prior stage of bone transport, all necrotic and infected bone and soft tissues are removed from the hardware completely, debrided thoroughly, and/or implanted with antibiotic-impregnated cement spacers to improve stability. The bacterial culture and antibiotic sensitivity test were conducted in exudation, to instruct the surgeon to apply appropriate postoperative antibiotics. Local tissue flaps or direct sutures were performed to reconstruct small soft tissue defects without tension, yet flap transfers or free skin grafts were applied to cover larger defects.

At the bone transport surgery stage, the Ilizarov device with a hinge movement system and 3 or 4 rings was constructed to simplify the operation before the operation, depending on the calculated angle deformity and limb length difference. The patients were performed under general anesthesia and placed in a supine position on the radiolucent operating table. The Kirschner wire (diameter, 1.8 mm) and a half-needle (diameter, 6.5 mm) were used to place the ring perpendicular to the long axis of the proximal and distal segments of the bone defect, and the X-ray was conducted to confirm their position. The distance was maintained up to 1.5-2.0 cm between the inner edge of each ring and the skin, and keep a distance of about 5 cm between the osteotomy site and the adjacent ring. Besides, two or three crossed olive wires (tension, 110–130 Nm) parallel to the joint line to fix each ring near the knee joint or ankle joint. When the distal bone fragment less than 2.0 cm, the ring adjacent to the ankle should be fixed at the level of the calcaneus or talus to provide sufficient stability for the whole frame. Simultaneously, excessive compression of the ankle joint surface should be avoided as much as possible to prevent further degeneration of the cartilage on the joint surface even if the joint was fused. One or more metal spicules were then inserted to ensure that the device has sufficient stability. Two metal pins were inserted so perpendicular to the mechanical axis of the tibia to fix the two rings near the osteotomy. Further, the required retraction of the proximal or distal skin was obtained to avoid skin tension and ensure the correction of the deformity and bone transport. Additionally, two hinges and a motor unit were adjusted to place on the bisector of the deformed convexity, and the bisector divides the true angle of the deformity into two equal halves, which was to ensure that the deformity correction period was normal. Last, a percutaneous osteotomy was performed on the fibula at the apex of the deformity using minimally invasive techniques.

### Data collection

The basic demographics were obtained by standardized self-administered questionnaires at the beginning of in-patient treatment and complemented by extracting relevant data from hospital records, included: age, gender, body mass index (BMI = weight (kg) /height (m^2^)), location of bone defect (proximal, middle and distal), comorbidities (such as diabetes, hypertension, and osteoporosis) and the history of metal allergy and glucocorticoid intake. Patients’ preoperative conditions such as the duration of disease, the American Orthopaedic Foot & Ankle Society (AOFAS) ankle-HF scale scores, defect size (DS), the type of bone transport (single level and double level) were documented as well.

Postoperative data which included the bone union time, external fixator time (EFT/ET), and external fixator index (EFI) was recorded. EFT referred to the time spent before removing the external fixator. EFI is defined as the ratio of EFT days to the distraction regenerate length (cm). Imaging evaluations were performed every 2 weeks during the distraction phase and monthly during the consolidation phase. All patients were followed up closely for at least 2 years after the external fixator was removed.

### Potential risk factors

Continuous variables included age, defect size (DS), the duration of hospitalization, the AOFAS ankle-HF scale scores, the duration of the bone union, external fixator time (EFT/ET), and external fixator index (EFI). And gender, body mass index (normal weight = BMI < 25 kg/m^2^, overweight = BMI > 25 kg/m^2^), location of bone defect (proximal, middle, and distal), comorbidities such as diabetes, hypertension, and osteoporosis (yes or no), the history of metal allergy (yes or no), glucocorticoid intake (yes or no), and the type of bone transport (single level and double level) were attributed to the categorical variables.

### Postoperative management

The pin tracts were covered by the gauze and rubber plugs and replaced every 2 to 3 days to keep them dry and prevent infection. Patients were encouraged to perform isometric muscle and joint range of motion (ROM) exercises within pain tolerance on the first day after surgery. Besides, standing and walk for at least 2 h per day was recommended, and switch from partial weighting to full weight-bearing as soon as possible. According to the results of exudation culture and antibiotic sensitivity test, select sensitive antibiotics for intravenous application for at least 3 weeks or until the erythrocyte sedimentation rate (ESR) and C-reactive protein (CRP) levels return to normal.

Slow and stable distraction was commenced on postoperative day 8 with the rate of 0.25 mm per day at the apex of the bone defect and continued in 3 or 4 equal increments until the required angle correction was reached. Subsequently, the distraction rate was maintained at 0.25 mm per day until the bone connection was reached. X-rays of the local bones and the entire length of the lower limb were conducted regularly to evaluate the degree of correction, distraction, and consolidation. Consolidation was considered sufficient when 3 of the 4 visible cortexes in the anteroposterior and lateral radiographs were visible bridging callus formation and the tenderness at the osteotomy site and pain during weight-bearing without connecting rods. After confirming the consolidation, the external circular frame was removed under local anesthesia in the outpatient clinic.

All radiographs were evaluated by at least two senior orthopedic doctors and two residents. The radiographs and clinical symptoms were then diagnosed as ankle OA correctly, based on the original version of the K&L scale (Table 1). Additionally, the presence or absence of dock site nonunion, pin tract infection, axial deviation, as well the presence and localization of osteophytes in all three ankle compartments: superior, medial tibiotalar, and talofibular were evaluated. The osteophytes were classified as absent, doubtful significance, or present. All patients were assessed by two orthopaedic surgeons and the American Orthopaedic Foot & Ankle Society (AOFAS) ankle-HF Scale scores were documented for the operated ankle at the follow-up.

**Table 1:** Original K&L scale

K&L scale	
1	Minute osteophyte of doubtful significance
2	Definite osteophyte, joint space unimpaired
3	Moderate diminution of joint space
4	Joint space greatly impaired, subchondral sclerosis

### Statistical analysis

Statistical analysis was performed with the SPSS 23.0(IBM Corp, USA). Continuous variables, such as age, weight, and height (BMI = weight (kg) /height (m2)), defect size (DS), the duration of hospitalization, the AOFAS ankle-HF scale scores, the duration of the bone union, external fixator time (EFT/ET), and external fixator index (EFI), were analyzed by independent-samples T-tests and expressed as the mean and standard deviation. And the categorical variables included sex, location of bone defect (proximal, middle, and distal), comorbidities (such as diabetes, hypertension, osteoporosis) and the history of metal allergy and steroid hormone intake and the type of bone transport (single level and double level), were analyzed by the Mann-Whitney U test, expressing as the number. Besides, comparing clinical scores in patients with K&L grades < 2 group vs. K&L grades > 2 group to evaluate clinical significance. A statistically significant difference was set at P < 0.05.

The variable with a P value of 0.05 or less in the Mann-Whitney U test or T-test was entered to in the multivariate logistic regression model the relationship between the explanatory variable and the ankle OA and control the potential confusion of any included variables. The odd ratio provides a 95 % confidence interval and P value. A P value of less than 0.05 was considered statistically significant. The cumulative number of risk factors was determined for each patient (possible range from 0 to 5) and evaluated the incidence of ankle OA.

## Results

There were 236 bone defects in 236 patients corresponding to the inclusion criteria with a mean defect size of 5.9 cm (4.1–7.2 cm) and operated upon during the research period. Of these patients, no one died, refused, or did not participate in the follow-up. Out of 236 patients, 49 had an additional surgery for ankle OA after the Ilizarov technique treatment for bone defects (average follow-up time 2.1 years, range 1.6–4.2 years) between May 2008 and January 2018. The incidence of postoperative ankle OA was 20.8 %, with nineteen patients classified as K&L grade 3 and seven patients as grade 4. In detail, subtalar joint arthritis in 17 cases (34.6 %), talonavicular joint arthritis in 12 cases (24.4 %), and calcaneocuboid joint arthritis in 18 cases (36.7 %). Fortunately, ankle fusion was achieved in all patients with ankle OA after additional correction surgery, in both clinical and radiographic criteria. Pin tract infection occurred in 18 cases, delayed union on docking site was presented in 7 cases, axial deviation appeared in 12 cases, poor regenerate consolidation was observed in 1 case, and none refracture on docking site after fixator removal occurred. Eighteen pin tract infections were resolved by local saline washes, occlusive dressings, and oral antibiotic therapy. Twelve axial deviations were corrected by adjusting the external fixator manually. Seven delayed unions on docking sites and one poor regenerate consolidation were recovered through surgical treatment of autologous bone grafts.

Comparison of the demographic data between those patients seen at follow-up (Table 2) revealed that there was no significant difference concerning the location of bone transport, metal allergy, and bone union time from the original cohort. In the univariate logistic regression analysis, the following were associated significantly with a higher incidence of ankle OA: age > 45years, male, BMI > 25, double level bone transport, diabetes, hypertension, osteoporosis, glucocorticoid intake, duration of disease > 24 months, EFI > 50days/cm, AOFAS ankle-HF scale scores < 50 (Table 3). DS > 5 cm and EFT > 300 days were not in a significant association with ankle OA. The incidence greater than 50 % was found in patients with BMI > 25 (51.1 %), male (53.1 %), age > 45 ears (56 %), diabetes (57.1 %), double level bone transport (59.1 %), as well as osteoporosis (85.7 %). Male, BMI > 25, diabetes, osteoporosis, and AOFAS ankle-HF scale scores in poor were associated independently with the ankle OA in the multivariate logistic regression analyses and constituted the final model as presented in Table 4.

**Table 2:** Baseline characteristics of patients

	All interventions (*n* = 236)	Ankle OA (*n* = 49)	Not ankle OA (*n* = 187)	*p* value
Male (%)	199(84.3 %)	26(53.1)	173(92.5)	< 0.001
Age, mean(± SD)	47.21(± 8.16)	54.73(± 3.43)	47.44(± 8.11)	< 0.001
BMI (%)				< 0.001
<25	76(32.2)	21(42.9)	55(29.4)	
>25	160(67.8)	28(51.1)	132(70.6)	
Location (%)				0.943
proximal	44(18.6)	9(18.3)	35(18.7)	
middle	89(37.7)	19(38.7)	70(37.4)	
distal	103(43.6)	21(42.8)	82(43.9)	
Type (%)				< 0.001
Single-level	107(45.3)	20(40.8)	78(41.7)	
Double-level	129(54.7)	29(59.1)	109(58.3)	
Diabetes yes (%)	91(38.6)	28(57.1)	63(33.7)	0.003
Hypertension yes (%)	76(32.2)	13(26.5)	63(33.7)	0.002
Osteoporosis yes (%)	97(41.1)	42(85.7)	55(29.4)	< 0.001
Metal allergy yes (%)	37(15.7)	22(44.9)	15(0.08)	0.548
Glucocorticoid intake yes (%)	116(49.2)	24(48.9)	92(49.2)	0.027
Duration of disease, mean(± SD)	25.26(± 6.88)	26.82(± 5.25)	18.68(± 1.57)	< 0.001
DS, mean(± SD)	5.90(± 1.59)	7.70(± 0.56)	5.12(± 0.58)	< 0.001
Bone union time, mean(± SD)	16.31(± 1.51)	11.42(± 1.72)	7.85(± 0.74)	0.929
EFT, mean(± SD)	253.94(± 59.00)	348.34(± 6.64)	302.02(± 4.43)	< 0.001
EFI, mean(± SD)	68.63(± 6.12)	44.49(± 7.24)	56.98(± 5.92)	0.040
AOFAS	65.96(± 12.82)	48.79(± 8.33)	68.54(± 8.91)	< 0.001

*AOFAS* the American Orthopaedic Foot & Ankle Society (AOFAS) ankle-HF scale scores; *DS* defect size; *EFT* external fixation time; *EFI* external fixation index

**Table 3:** Univariate logistic regression analysis of risk factors for ankle OA

Factor	Odds ratio (95 % CI)	Standard error	*P* value
Age > 45years	2.29(0.63–0.87)	0.922	0.032
Male	0.54(0.18–0.63)	0.560	< 0.001
BMI > 25	1.34(0.32–0.56)	0.448	< 0.001
Double-level^a^	3.79(1.51–9.53)	0.470	0.005
Diabetes	0.31(0.13–0.75)	0.455	0.009
Hypertension	0.42(0.17–0.42)	0.437	0.046
Osteoporosis	1.58(0.21–1.5)	0.506	< 0.001
Glucocorticoid intake	0.33(0.13–0.8)	0.451	0.014
Duration of disease > 24 months	0.53(0.31–0.52)	0.274	0.002
DS > 5 cm	1.01(0.56–0.71)	0.297	0.675
EFT > 300days	1.41(0.51–0.69)	0.118	0.999
EFI > 50days/cm	3.17(0.97–1.09)	0.931	0.015
AOFAS < 50	1.26(1.18–1.34)	0.032	< 0.001

^a^Type of bone transport. AOFAS, the American Orthopaedic Foot & Ankle Society (AOFAS) ankle-HF Scale scores

**Table 4:** Multivariate logistic regression analysis of risk factors for ankle OA

Factor	Odds ratio (95 % CI)	Standard error	*P* value
Male	0.27(0.02–0.32)	0.993	0.003
BMI > 25	1.11(0.12–1.05)	1.241	0.005
Diabetes	0.128(0.17–0.96)	1.027	0.045
Osteoporosis	0.11(0.01–0.11)	1.152	< 0.001
AOFAS < 50	0.69(0.58–0.81)	0.084	< 0.001

*AOFAS* the American Orthopaedic Foot & Ankle Society (AOFAS) ankle-HF Scale scores

**Fig. 1:**
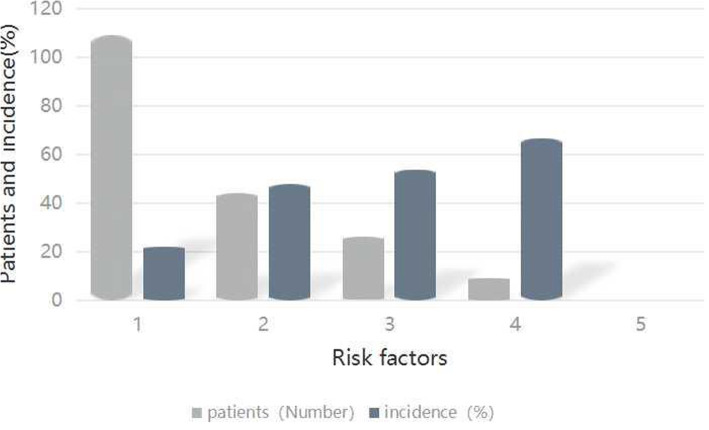
Incidence of ankle OA according to the number of risk factors

Regarding the accessorial outcome of ankle OA of the ankle, 9 patients (18.3 %) presented with an AOFAS ankle-HF scale score < 50 and a K&L grade 3–4. Additionally, the incidence of ankle OA per individual per risk factor was presented (Table 5) and increased substantially in the presence of progressively more risk factors (Fig. 1). The incidence of ankle OA was lower than 30 % in patients with one risk factor, and even zero with the none risk factor. However, those with two, three, and four risk factors increased to 47.7 %, 53.8 %, and 66.7 %, respectively.

**Table 5:** Incidence of PTOA according to the number of risk factors present

Risk factors(n)^a^	Patients (n) per risk factor category	Incidence of ankle OA
0	0	0(0 %)
1	109	24(22 %)
2	44	21(47.7 %)
3	26	14(53.8 %)
4	9	6(66.7 %)
5	-	-

^a^To categorize patients whether at risk or not, the continuous risk factors were dichotomized: age > 45years vs. age < 45years, male vs. female, BMI > 25 vs. BMI < 25, double level vs. single level, diabetes vs. not diabetes, hypertension vs. not hypertension, osteoporosis vs. not osteoporosis, glucocorticoid intake vs. not glucocorticoid intake, duration of disease > 24 months vs. duration of disease < 24 months.

## Discussion

Our study characterized the incidence of ankle OA and its correlations with clinical data, which was assessed by the K&L scale and AOFAS scale. [12, 19]. It was also noteworthy that ankle pain, stiffness, and physical function disability increased significantly with K&L grades and AOFAS ankle-HF Scale scores. After an average of 2.1 years in receiving the reconstruction operation of the tibial defect with an external circular fixator of the Ilizarov technique, forty-nine (20.8 %) of 236 patients developed ankle OA. This differs from the results described by Morasiewicz et al. [7] for external fixation in the treatment of ankle OA since their subjects were patients who did not receive bone transport for critical bone defects, and the sample size was insufficient. However, the patients in our cohort developed ankle symptoms after bone transport procedure, then the incidence of ankle OA and the healing rate after fusion surgery were not consistent with most similar studies [20–23]. Compared with other methods, the advantages of the external circular fixator also included rigid immobilization, resistance to shear and torsional stresses, axial loading with the ability to restore early weight-bearing status, and manageability of large soft tissue with bone defects [24].

Unfortunately, other complications occurred in patients as well, including pin tract infection(18 cases),delayed union on docking site(7 cases), axial deviation(12 cases) and poor regenerate consolidation(1 case), which are consistent with the complications related to the Ilizarov technique described by Liu et al. [25]. These complications may be one of the reasons for such a high incidence of ankle OA in our cohort of patients. According to the published study, the incidence of ankle OA was increased in patients who obtained ankle instability and premature functional exercise [7, 12, 13, 16, 26, 27]. Complications of bone defect reconstruction using the Ilizarov technique had been proven to increase the chance of ankle instability and the compression of the joint surface [20]. For instance, poorly controlled pin tract infection may lead to pin tract loosening, which increases the additional treatment time, the rate of osteoporosis, or delay the connection of the docking site further, and promote the incidence of ankle OA lastly. In this study, the same phenomenon was noticed that the patients who experienced more complications, which postpone the period of whole treatment, gained the ankle OA easier. Thus, it’s of great importance for the prevention of ankle OA to keep the pin tract clean and dry, underwent X-rays radiograph to observe the position of bone transport regularly, and make timely management for the other complications.

A multivariate analysis was conducted among the baseline data of ankle OA patients and the variables with statistical significance were analyzed by logistic regression analysis. Subsequently, age > 45years, male, BMI > 25, double level bone transport, diabetes, hypertension, osteoporosis, glucocorticoid intake, duration of disease > 24 months, EFI > 50days/cm, AOFAS ankle-HF scale scores < 50 were associated with a greatly increased risk of ankle OA as defined by the K&L scale. The incidence of ankle OA was 50–70 % in the existence of three or more risk factors. With the help of meticulous multiple logistic regression statistical analysis, independent risk factors for the development of ankle OA were associated with the BMI > 25, male, diabetes, osteoporosis, AOFAS ankle-HF scale scores < 50.

Despite the changes and clinical symptoms of osteoarthritis assessed by radiography were controversial [28], a substantial association was found between pain, functional scores, and the appearance of radiographic ankle OA. In hip and knee, the strong relationship between clinical and radiography in a people-based cohort had been disclosed [29], yet uncommon in the ankle. In our observation, the potentially modifiable risk factors were BMI [13, 26, 30], and chronic diseases such as diabetes, hypertension, and osteoporosis, could be managed to reduce the probability of ankle OA effectively. Thus, patients should be messaged about these additional risks and instructed towards weight reduction and management of chronic diseases. The other reason for risk factors identification was to provide patients with recommendations regarding the future risk of ankle OA (individualized prediction) and match available treatment options to postpone poor prognosis.

Obesity (BMI > 25) had been certified as an independent risk factor for OA in previous studies [31]. In our cohort, the probability of ankle OA with different K&L levels in obese patients (OR1.34, CI0.32-0.56) was as high as 57.1 % after the tibia bone defect reconstruction surgery. The main reason for our consideration was that obesity increased the axial load and inflammatory response of the lower limb mediated by ankle alignment, which accelerated the process of cartilage degeneration [26, 31], and led to ankle OA further. Then joint damages were almost caused by the change of mechanics through altered surface alignment, forced distribution, and varying degrees of ongoing joint instability [32]. Besides, ankle OA was more likely to occur in male patients (OR0.54, CI0.18-0.63) over the age of 45(OR2.29, CI0.63-0.87) when treated with external circular fixators for bone defects in our study. According to our observation, this was probably the more opportunities they had to develop a significant smoking history, diabetes, alcohol abuse, and chronic diseases, as a result of decreased metabolism and excessive physical work than females. This was in agreement with Holzer et al.[16] findings of a higher incidence of OA in the presence of an associated older male.

The increasing prevalence of OA with age may be the result of cumulative exposure to various risk factors and biological changes [7, 27, 33]. Briefly, mechanical protective mechanisms, due to decreased muscle hypertrophic capacity in the elderly, this is likely to result in loss of joint protection with age during normal gait cycles. And a remarkable advance from the late 90 s was the description of cellular metabolic pathways affected by aging [33]. With the aging of the body, the metabolism and enzymatic reaction of various types of cells in the body, which nourishment of articular cartilage required, are inevitably reduced. Thus, age is a quite important risk factor for weight-bearing joint OA, like the ankle joint.

The published researches had revealed that the double level bone transport shortened the EFT and EFI greatly in the comparison of the single level bone transport [1, 2, 4, 34]. Similarly, the EFT and EFI of patients with double level bone transport in this study were lower than those of patients with single level. However, the phenomenon that the number of ankle OA in patients with double level bone transport (59.1 %) was more than that in patients with single level (40.8 %) was noticed. Afterward, there was a stronger correlation was confirmed by the univariate logistic regression analysis between double level bone transport and ankle OA (OR 3.79, CI1.51-9.53). Moreover, the DS is also the basic factor for choosing double-level bone transport, especially in the DS > 5 cm. Thus, our cognition for this was that double-level bone transport reduced EFI and the period of Ilizarov technique treatment, but it required stronger forces on the tibia and ankle to ensure the stability of the entire external circular fixator frame, which made the movement of the ankle more limited and aggravated the degeneration of articular cartilage. Given the above limitations of this technique and the lack of regenerative capacity of articular cartilage, it was not surprised that the double level bone transport was a responsible risk factor for the development of ankle OA [35, 36]. This had also been verified by studying complications of the Ilizarov technique which point to high rates of osteoarthritis development, even after surgical anatomic joint reduction [25, 37].

However, although mechanical overload was assuredly a risk factor in the development of ankle OA, it was insufficient on its own to explain why degradation of cartilage also occurred in non-overload affected areas of cartilage. For instance, osteoporosis (OR1.58, CI0.21-1.5) and chronic disease subjects, such as diabetes (OR0.31, CI0.13-0.75) and hypertension (OR0.42, CI0.17-0.42), seemed to sustain more severe types of ankle OA in our study. The majority of patients with osteoporosis in our cohort could be explained by the mechanism of bone defects caused by long-term post-traumatic osteomyelitis (> 24 months), which had been treated for sufficient time before bone transport was initiated. This may make a mess of the microenvironment for bone regeneration and soft tissue coverage, which then delayed docking site union and bone mineralization. In Hannah et al.[30] research, researchers found that natural changes were observed in chronic diseases patients’ ankle included structural change of type II collagen and a decreased capacity of chondrocytes to repair the damaged extracellular matrix, accompanied by the progression of diabetes and hypertension [27, 30]. The ankle OA was caused probably by this condition of local high glucose consistency and vascular resistance reduction of chondrocyte differentiation, then the potential cartilage regeneration was undermined. Clinical outcomes from this study may stand by that the chondrocytes in chronic disease patients’ cartilage may be less effective in repairing articular surface damage.

AOFAS ankle-HF Scale scores had been reported to have a greater capacity for diagnosing the degeneration of ankle function [19, 38]. Likewise, patients with high scores (OR1.26, CI1.18-1.34) had a greater risk of obtaining ankle OA in our observation. Therefore, despite the biological mechanism on ankle cartilage degeneration is complex, clinicians should pay more attention to the patients who acquired high scores and make good use of this accessorial tool.

Further, the relationship was also observed between the number of risk factors and risk categories according to logistic regression analysis based on the scale scores. In the presence of 3 or more risk factors, the risk of developing ankle OA appears to increase notably and continued to rise with the number of factors (Fig. 1). More than half of the patients (153 of 236) fell into two or fewer risk factors category with the occurrence rate of ankle OA of 22–47 %, the risk factors of 3 or more (35/236) were disclosed at a higher rate of 53–66 %.

Yet, certain limitations existed in our study. Firstly, given its retrospective nature, small sample size, and exclude the patients older than 60 years, caution should be taken regarding the interpretation of our analytical results. The actual rate of ankle OA could be higher (some ankle insidious pain may not be brought to the attention of patients). Secondly, insufficient postoperative management instruction could give rise to higher numbers of ankle OA due to the complex nature of the postoperative rehabilitation of the Ilizarov technique, and more confounding factors coming into play. Thirdly, to get control of the limitations of our study, further studies with larger sample size, longer follow-up period, and multicenter design should be designed.

## Conclusions

The study we performed for demonstrating the quantification of risk factors for ankle OA and their role allows one to determine the prognosis of a disease/outcome, which is important for both clinicians and patients. Almost 20.8 % of patients presenting within an average of 2.1 years following Ilizarov technique treatment using an external circular fixator are diagnosed with ankle OA. The double level bone transport, EFI > 50days/cm, age > 45years, osteoporosis, BMI > 25 are the top five relevant risk factors of ankle OA. Male, BMI > 25, diabetes, osteoporosis are the independent risk factors. The risk of ankle OA seems to continue to increase as the number of factors increases. Clinicians should be aware of these risk factors to figure out the patient’s risk condition for collaboration with a view to receiving favorable long-term results after the treatment.

## Data Availability

The datasets analyzed during the current study are available from the corresponding author on reasonable request.
